# The curious case of Kounis syndrome: exploring clinical manifestations and management in the presence of nonobstructive coronary arteries

**DOI:** 10.21542/gcsp.2024.14

**Published:** 2024-03-03

**Authors:** Darren Drittel, Dylan Deyar, Eric Boxer, Hussam Al Hennawi, Margaret Mack

**Affiliations:** 1 Thomas Jefferson University, Philadelphia, PA, USA; 2 Philadelphia College of Osteopathic Medicine, Philadelphia, PA, USA; 3 Department of Internal Medicine, Jefferson Abington Hospital, Abington, PA, USA

## Abstract

Kounis syndrome, an allergic hypersensitivity coronary disorder, is a rare but potentially life-threatening condition triggered by various allergens, including medications. We present the case of a 41-year-old male with no prior cardiac history, who developed Kounis syndrome following vancomycin administration for suspected cellulitis. The patient initially presented with rash, fever, and malaise, which progressed to chest discomfort associated with diaphoresis and elevated troponin levels. Diagnostic evaluations, including electrocardiographic changes and coronary angiography, confirmed a diagnosis of type I Kounis syndrome. This case adds to the limited literature on vancomycin-induced Kounis syndrome, and underscores the importance of considering this diagnosis in patients with myocardial damage following exposure to potential allergens.

## Background

Kounis syndrome (KS), first described in 1991, is an allergic hypersensitivity coronary disorder most commonly induced by food, medications, and environmental triggers^1^. The release of inflammatory mediators from mast cells and basophils causes coronary artery vasospasm and myocardial ischemia^1^. KS can be classified by the presence of an underlying cardiac history and/or prior intracoronary stent placement^2^. Risk factors for the development of KS include medical history of allergies, hypertension, smoking, diabetes, and hyperlipidemia. Abdelghany et al. found that chest pain occurred most frequently as a presenting symptom (86.8%), followed by anaphylaxis (53%) and rash (26.8%)^3^. KS affects all ages and sexes, although it is more frequent in men (74.3%), ages 51–60 (29.7%), and 60–70 (23.4%). Medications, such as penicillins and cephalosporins, frequently trigger this. The list of documented medication triggers of KS is extensive and includes antibiotics (penicillins, cephalosporins, metronidazole, clindamycin, clarithromycin), antivirals (oseltamivir, brivudine), fluconazole, lanzoprole, NSAIDs (ibuprofen, diclofenac), enalapril, estimazole, anesthetics (propofol, midazolam, etomidate), neuromuscular blockers (atracurium, rocuronium), anti-cancer drugs, and others^3^. The increasing prevalence of KS, along with the increasing discovery of new triggers, has led many experts to agree that KS has historically been underdiagnosed^4^. The most common vancomycin adverse effects are hypotension, nephrotoxicity, ototoxicity, and an infusion reaction consisting of upper body flushing (formerly known as “Red Man Syndrome”)^5^. There have been only three documented cases of vancomycin-induced Kounis Syndrome. Here, we report a case of Kounis syndrome secondary to vancomycin administration.

## Case presentation

A 41-year-old male, previously diagnosed with psoriasis and hidradenitis suppurativa following extensive gluteal and perianal surgical procedures with grafting, was admitted to the hospital because of a rash extending from his left flank down to his left thigh and accompanying symptoms of headache, general malaise, fever, and chills.

Upon arrival, the patient was hemodynamically stable and no anginal symptoms were reported. Notable physical findings included a well-demarcated, brightly erythematous rash with non-raised borders, extending from the lower back over the gluteal area down to the upper thigh. The affected skin was warm to the touch and exhibited associated flaking and peeling, without the presence of vesicles or pustules. There was no central clearing or satellite lesions.

**Figure 1.**
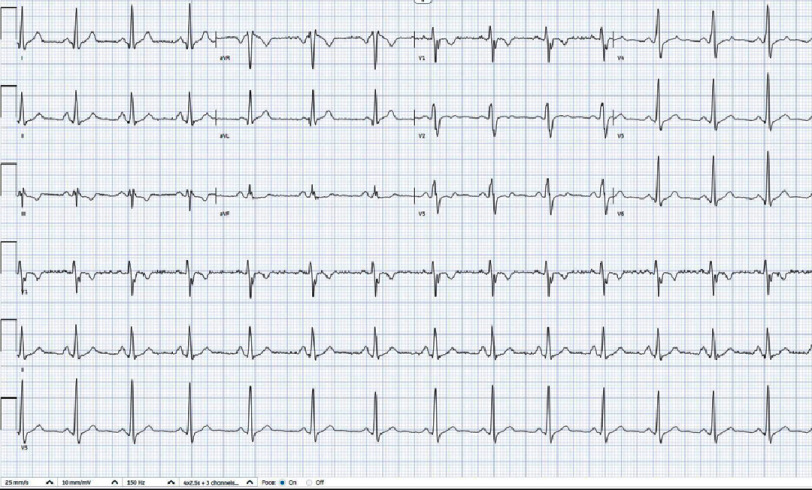
An electrocardiogram during admission showed normal sinus rhythm.

The initial treatment approach involved administering intravenous vancomycin (2 g) for suspected cellulitis. The patient had a fever of 100.4°F on admission but did not display leukocytosis. Laboratory results revealed normal creatinine levels, platelet counts, and a markedly elevated erythrocyte sedimentation rate (ESR). Urinalysis revealed only two white blood cells. Testing for SARS-CoV-2 and influenza yielded negative results, and a chest X-ray displayed no active disease. Additionally, a CT abdominal/pelvic scan with IV contrast revealed no acute intra-abdominal or pelvic abnormalities, abscesses, or hepatic steatosis. Electrocardiography (ECG) showed normal sinus rhythm (Figure 1).

**Figure 2.**
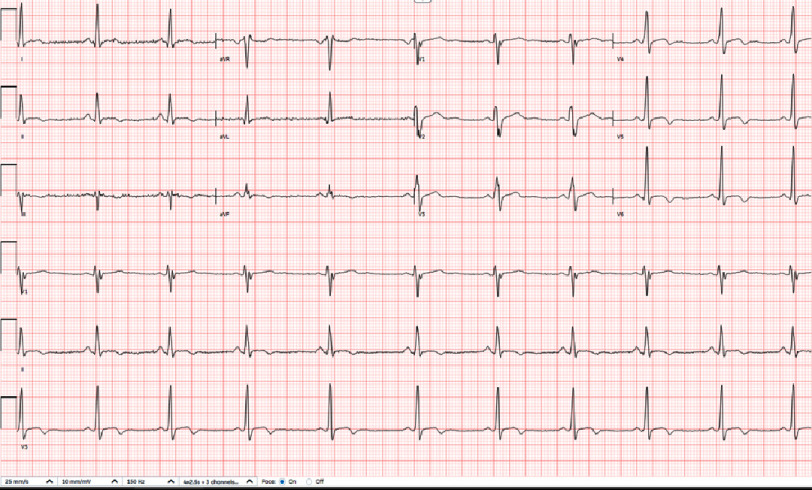
Electrocardiogram showed nonspecific intraventricular conduction delay (IVCD) and T-wave inversions in the inferior lateral leads.

The patient received a total of three doses of IV vancomycin (2 g loading dose followed by 1.5 g every 12 h). Given the improvement in the patient’s condition and unremarkable infectious workup, vancomycin was discontinued and switched to intravenous cefazolin. Twelve hours after the last dose of vancomycin, the patient complained of chest discomfort lasting approximately one hour associated with diaphoresis, nausea, and headache. He described the pain as dull and centrally located with a severity rating of 5/10. The discomfort was not associated with shortness of breath or palpitations. Pain was not affected by inspiration or postural changes. Sublingual nitroglycerin 0.4 mg provided some relief. Elevated levels of inflammatory markers were detected. His blood pressure was 115/81 mmHg, and electrocardiography (EKG) revealed nonspecific intraventricular conduction delay (IVCD) and T-wave inversions in the inferior lateral leads (Figure 2).

**Figure 3.**
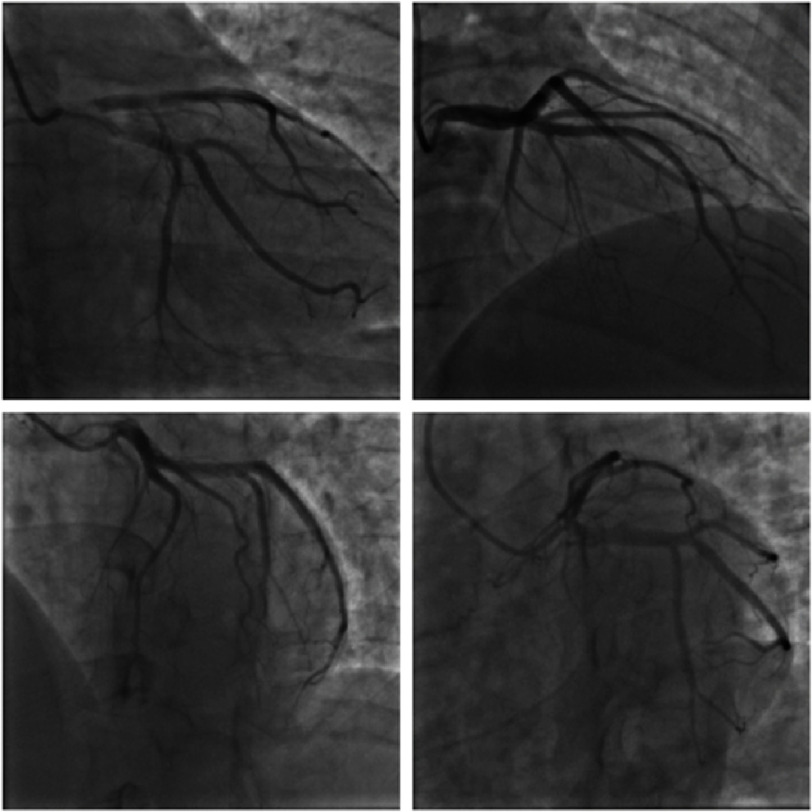
Coronary angiography showing patent epicardial coronary arteries with normal LVEDP (left ventricular end-diastolic pressure).

His initial troponin level was 30 ng/L, which later increased to 199 ng/L. The patient received a loading dose of aspirin 325 mg, followed by aspirin 81 mg daily, and was placed on an intravenous heparin drip. Subsequent transthoracic echocardiography (TTE) yielded relatively unremarkable results, indicating an ejection fraction (EF) of 50–55%. Left heart catheterization (LHC) revealed no obstructive coronary artery disease (Figure 3). Notably, the patient did not experience chest pain recurrence.

## Discussion

In this report, we describe a case of KS in a 41-year-old male with suspected cellulitis who developed acute coronary syndrome (ACS) following vancomycin administration. KS is defined as a hypersensitivity reaction that affects the coronary arteries and leads to acute coronary events such as coronary spasm, acute myocardial infarction, and stent thrombosis. Various triggers, including certain medications, have been identified as the causes of KS. Antibiotic use has been linked to several KS cases. Although drugs have caused the majority of antibiotic-induced KS cases in the beta-lactam class, there are rare case reports of KS caused by vancomycin^6^. To the best of our knowledge, this is the fourth reported case of vancomycin-induced KS.

Although the exact mechanism of KS is unknown, the most widely accepted etiology involves mast cells, which release inflammatory mediators (histamine, chemokines, tryptase, and arachidonic acid products, such as leukotrienes) in response to allergic stimuli^7^. These mediators exert various effects on the cardiovascular system. For example, histamine induces vasoconstriction of the coronaries and promotes activation of the coagulation cascade, whereas tryptase causes plaque rupture and the formation of intravascular thrombi^6^.

Three subtypes of KS have been previously described^8^. Type I variant is seen in patients without any underlying coronary artery disease and involves inflammation-mediated coronary artery vasospasms. Type II variant is seen in patients with pre-existing atheromatous disease in which the allergic/inflammatory insult results in coronary artery vasospasms and/or plaque rupture. Type III variants are observed in patients with drug-eluting stents, in which immune complex deposition results in stent or coronary thrombosis^9^.

Our patient was ultimately diagnosed with KS, type I, based on several factors, including the temporal relationship between the initiation of IV vancomycin and the onset of chest pain, laboratory and electrocardiographic evidence of myocardial damage demonstrated by elevated troponins and T wave inversions, resolution of symptoms and ECG findings following conservative anti-ischemic treatment and cessation of the antibiotic, and absence of coronary artery abnormalities on cardiac catheterization.

In both this case and a similar case described by Gagnon et al., symptoms did not develop until the third dose of IV vancomycin was administered^10^. This is in contrast to two other cases of vancomycin-induced KS in which symptoms developed within 5 min of receiving the initial dose, suggesting that clinical manifestations of KS are dosing-dependent and may depend on individual differences in immune response or metabolic processing of the drug^6,11^.

Despite differences in temporal onset, the symptoms experienced by our patient were similar to those described in a similar case report of a 57-year-old man with vancomycin-induced KS who also presented with chest pain, diaphoresis, and headache^11^. In contrast, a more severe presentation was observed in an 83-year-old female with vancomycin-induced KS who presented with unresponsiveness and signs of respiratory distress and hemodynamic instability shortly after vancomycin infusion^6^.

Common allergic/anaphylactic symptoms such as urticaria, pruritus, angioedema, congestion, and wheezing were absent across all case reports of vancomycin-induced KS, including this present study^6,10,11^. This underscores the significance of recognizing that KS, despite its underlying etiology as a mast-cell-driven type I hypersensitivity reaction, can still be a diagnostic possibility, even in the absence of typical allergic symptoms.

Diagnostic testing results differed between our patient and the other three cases of vancomycin-induced KS. For example, serum troponin-I levels were elevated to 199 ng/L in our patient’s case and 12,230 ng/L in a case report described by Gagnon et al., but remained undetectable in the other two case reports.^10^ Additionally, ECG changes in our case showed nonspecific intraventricular conduction delay (IVCD) and T-wave inversions in the inferior lateral leads, while the other three cases demonstrated ST elevations on ECG. In all four cases, ECG tracings returned to baseline once vancomycin was stopped. KS has been associated with various electrocardiographic changes, making the results nonspecific^7^. What’s more important is the presence of any ECG alteration, rather than the specific nature of the ECG change.

This case report contributes to the limited body of knowledge on vancomycin-induced Kounis syndrome. To the best of our knowledge, this is the fourth case report documenting vancomycin-induced Kounis syndrome. Additionally, our study is the first to compare and contrast the clinical presentation of vancomycin-induced Kounis syndrome, highlighting the heterogeneous manifestations of this condition (Table 1). While this condition may lack clear-cut diagnostic features, it should still be considered a potential diagnosis in individuals with signs of myocardial damage who have been exposed to drugs or other substances capable of triggering hypersensitivity reactions.

**Table 1:** Overview of vancomycin-induced Kounis Syndrome cases reports from the literature.

**Authors** [***Reference #***]	**Age and Sex**	**Presenting Complaint, Primary Diagnosis**	**Past Medical History**	**Drug Exposure**	**Post-Exposure Presentation**	**Serum Troponin-I**	**ECG Findings**	**KS Treatments**
Present Study	41, M	Rash, cellulitis	Hidradenitis suppurativa, psoriasis, tobacco use	IV Vancomycin (2 g)	Chest discomfort, nausea, diaphoretic, headache	199 ng/L	T-wave inversions in inferior lateral leads, IVCD	Sublingual nitroglycerin (0.4 mg), oral aspirin (325 mg and 81 mg), IV heparin (11.3 units/kg/hr)
Martinez et al.^6^	83, F	Knee pain, infected joint	Hypothyroidism, HTN, AVR, HLD, BTKA	IV Vancomycin (15 mg/kg)	Unresponsive, agonal respiration, thready pulse	Undetectable (<0.017 ng/mL)	ST elevation in inferior-lateral leads, reciprocal ST changes in precordial leads	IV diphenhydramine (50 mg)
Leibee et al.^12^	57, M	Toe pain, osteomyelitis	PAD, DM, HTN, tobacco use	IV Vancomycin (15 mg/kg)	Diaphoretic, headache, chest pain	Undetectable (<0.04 ng/mL)	ST-segment elevation and hyperacute T waves in the inferior leads, reciprocal ST segment depression in high lateral leads	IV normal saline (1L), IV diphenhydramine (50mg q20 min), oral aspirin (325 mg q20 min),^a^
Gagnon et al.^10^	32, M	Inguinal abscess, cellulitis	Migraines, ADHD, toba ohh cco use	IV ceftriaxone; Oral TMP-SMX and metronidazole; IV Vancomycin (2 g loading dose,1.5 g q8h)	Chest pressure, nausea, right-arm paresthesia	12,230 ng/L	ST-segment elevation in the inferior leadhout reciprocal changes	Nitroglyceri, amlodipine (5 mg)^a^

**Notes.**

IVCD Intraventricular conduction delay HTN hypertension AVR Aortic valve replacement HLD hyperlipidemia BTKA Bilateral total knee arthroplasty PAD Peripheral artery disease DM diabetes mellitus ADHD Attention deficit hyperactive disorder TMP-SMX Trimethoprim-sulfamethoxazole q8H every 8 hours

a Vancomycin infusion stopped.

## Conclusion

This report highlights the heterogeneous clinical presentation of vancomycin-induced KS and the need to consider this diagnosis in patients with myocardial damage who have been exposed to potential allergens. While diagnostic criteria require further refinement, increased awareness and prompt diagnosis can aid in the timely treatment of this potentially life-threatening condition. Further research with larger sample sizes is warranted to establish clear diagnostic criteria and to guide physicians in managing KS effectively.
